# The Autophagy in Osteoimmonology: Self-Eating, Maintenance, and Beyond

**DOI:** 10.3389/fendo.2019.00490

**Published:** 2019-07-23

**Authors:** Lan Xiao, Yin Xiao

**Affiliations:** ^1^Key Laboratory of Oral Medicine, Guangzhou Institute of Oral Disease, Stomatology Hospital of Guangzhou Medical University, Guangzhou, China; ^2^Institute of Health and Biomedical Innovation, Queensland University of Technology, Brisbane, QLD, Australia; ^3^The Australia-China Centre for Tissue Engineering and Regenerative Medicine (ACCTERM), Queensland University of Technology, Brisbane, QLD, Australia

**Keywords:** osteoimmunology, bone remodeling, autophagy, immunomodulation, stem cell

## Abstract

It has been long realized that the immune and skeletal systems are closely linked. This crosstalk, also known as osteoimmunology, is a primary process required for bone health. For example, the immune system acts as a key regulator in osteoclasts-osteoblasts coupling to maintain the balanced bone remodeling. Osteoimmunology is achieved through many cellular and molecular processes, among which autophagy has recently been found to play an indispensable role. Autophagy is a highly conserved process in eukaryotic cells, by which the cytoplasm components such as dysfunctional organelles are degraded through lysosomes and then returned to the cytosol for reuse. Autophagy is present in all cells at basal levels to maintain homeostasis and to promote cell survival in response to cellular stress conditions such as nutrition deprivation and hypoxia. Autophagy is a required process in immune cell activation/polarization and osteoclast differentiation, which protecting cells from oxidative stress. The essential of autophagy in osteogenesis is its involvement in osteoblast differentiation and mineralization, especially the role of autophagosome in extracellular calcium transportation. The modulatory feature of autophagy in both immune and skeleton systems suggests its crucial roles in osteoimmunology. Furthermore, autophagy also participates in the maintenance of bone marrow hematopoietic stem cell niche. The focus of this review is to highlight the role of autophagy in the immune-skeleton interactions and the effects on bone physiology, as well as the future application in translational research.

## Introduction

The skeletal bone is a dynamic tissue with a life-long continuous renovation termed bone remodeling (1). This remodeling consists of bone resorption and formation and plays a fundamental role in the maintenance of bone homeostasis (2). Bone remodeling is kept in balance under physiological conditions, as the amount of bone resorption equals to that of formation (3). This balance is achieved via sophisticated regulations originated from the immune system (4). The link between the immune and skeleton systems has been identified for almost fifty years and termed as “osteoimmunology” (5). Further research into osteoimmunology has recognized the complex mutual regulations between immune cells and bone cells, that at one level, immune response determines the balance of bone remodeling, whereas on another level, bone cells mediate the polarization and function of immune cells (3, 4, 6). This interaction consists of multiple factors such as cytokines, receptors, signaling pathways (4); and it has been recently indicated that autophagy plays elementary roles in both immune (7) and skeletal (8) systems. Autophagy is defined as the delivery of cytoplasmic materials to the lysosome in animal cells or the vacuole in plant and yeast cells (9). Especially in eukaryotic cells, autophagy plays a key role in homeostasis maintenance (10, 11). Moreover, autophagy promotes cell survival in response to stress conditions such as nutrition deprivation and hypoxia (12). Autophagy is required in the differentiation of osteoclast and osteoblast (13, 14); meanwhile, it participates in the immune cell polarization/function and therefore regulates immune response (7, 15), suggesting a complex and rather intriguing role in osteoimmunology. This review highlights the effects of autophagy in the immune-skeleton interactions and proposes the regulation of autophagy for future application in bone regeneration.

## Autophagy Mechanism

There are mainly three types of autophagy, known as macroautophagy, microautophagy, and chaperone-mediated autophagy (9). The current review will focus on macroautophagy (hereafter referred to as autophagy), a highly conserved “self-eating” lysosomal degradation pathway in eukaryotic cells to clear intracellular waste (10, 11). Autophagy initiates with the sequestration of cytoplasmic organelles within double-membrane vesicles known as autophagosomes, which then fuse with lysosomes to form autolysosomes to degrade or recycle the autoghagic contents, such as damaged organelles, intracellular pathogens, glycogens, lipids, and nucleotides proteins (9, 16). Concomitantly, the cytosolic form of microtubule-associated protein 1A/1B-light chain 3 (LC3-I) is converted to form LC3-phosphatidylethanolamine conjugate (LC3-II), which is attached to the autophagosome membrane and then degraded (17, 18). This conversion from LC3-I to LC3-II is identified as one of the autophagy hallmarks. On the other hand, microautophagy is defined as the direct engulfment of small cytoplasmic portions by inward deformation of the lysosomal or late endosomal membrane (9, 19). Chaperone-mediated autophagy does not require membrane reorganization: the substrate proteins containing a KFERQ-like pentapeptide are selectively targeted by cytosolic heat shock cognate 70 (Hsc70) protein, then directly translocated into the lysosomal lumen (9, 20).

Autophagy is maintained at basal levels in all cell types, which plays a “quality control” role to maintain cellular homeostasis (11). On the other hand, autophagy is induced in response to stress conditions such as nutrient deprivation, oxidative stress, hypoxia and infection, which is one of the main strategies to promote cell survival (12, 21). Autophagy facilitates the “recycle” of cellular components and therefore provides energy for cells under starvation (22). Another important function of autophagy is to scavenge malfunctioning/damaged proteins and organelles (22). For example, autophagy-mediated clearance of damaged mitochondria, also termed as “mitophagy,” inhibits reactive oxygen species (ROS) accumulation and thereby protecting cell from oxidative stress and apoptosis (15, 23). This has been identified as a required process during bone cell differentiation and immune cell polarization, making a central role in osteoimmunology.

## Autophagy in the Skeletal System

### General Bone Biology

As the main constituent of the vertebrate skeletal system, bone serves as supporters and protectors of organs in the body (24). Bone consists of cellular components such as osteoclast, osteoblast, and osteocyte, as well as collagen, osteoid and inorganic mineral deposits (25). Osteoclast and osteoblast are the major players in bone remodeling (26). Originated from the hematopoietic stem cells (HSCs), osteoclast is considered as the major cell type responsible for bone resorption (25). The macrophage–monocyte lineage-derived osteoclast precursors fuse with each other to form a giant, multinucleated cell—the osteoclast (27). On the other hand, the mesenchymal stem cells (MSCs)-derived osteoblast is the major bone formation cell (25), which builds the osteoid matrix and eventually differentiate into osteocyte, the most abundant cell type in bone (28). The fact that osteoclastogenesis is dependent on osteoblast-derived receptor activator of nuclear factor factor-kappa B ligand (RANKL) (29, 30), suggests “coupling” between osteoclast and osteoblast, therefore linking osteoclastogenesis to osteogenesis (31). RANKL binds with receptor activator of nuclear factor factor-kappa B (RANK) on osteoclast-precursors, therefore initiating the differentiation of osteoclast. On the other hand, osteoblast produces osteoprotegerin (OPG), a decoy receptor of RANKL, to interrupt osteoclastogenesis (32). Hence, the balance between RANKL and OPG determines the outcome of bone remodeling (33). Furthermore, osteoblast secrets other factors to regulate osteoclastogenesis and osteogenesis in a paracrine or endocrine manner (34). For example, osteoblast is one of the major source of macrophage colony-stimulating factor (M-CSF) —a major factor for osteoclast differentiation—in the bone microenvironment (31, 35–37). Osteoblast-derived semaphorin 3A (Sema3A) and Wnt16 have been found to reduce osteoclastogenesis via interrupting the RANKL-RANK signaling (38, 39), while osteoblast-originated Wnt5 induces osteoclast differentiation by enhancing RANK expression in osteoclast-precursors (40, 41). Osteoblast also produces factors such as vascular endothelial growth factor A (VEGF-A) to induce osteogenesis (34, 42). Beside osteoblast, osteocyte is considered as another critical producer of RANKL, which also produces sclerostin (SOST) to reduce osteogenesis, therefore acting as the orchestrator of bone remodeling (43–46).

### Autophagy in the Differentiation/Function of Osteoclast, Osteoblast, and Osteocyte

**Figure 1:**
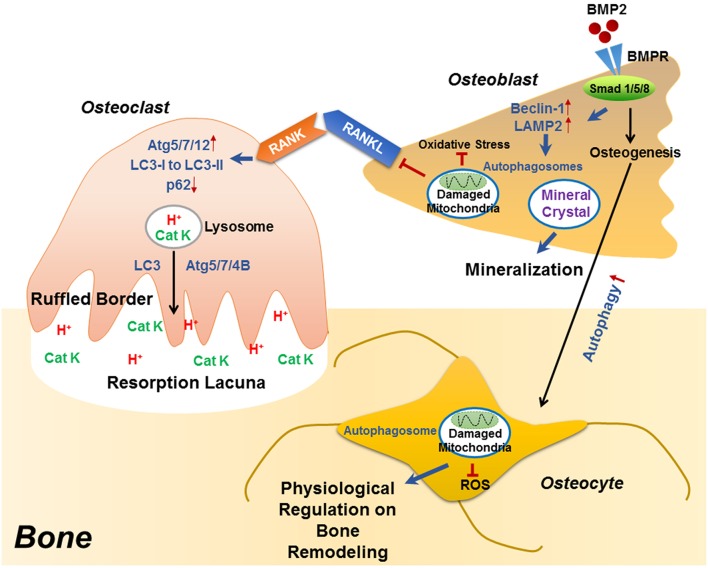
The role of autophagy in the differentiation/function of osteoclast, osteoblast and osteocyte. During RANKL-RANK induced osteoclast differentiation, the protein levels of ATG5/7/12 are increased, accompanied with enhanced conversion from LC3-I to LC3-II and p62 degradation, which plays an essential role in the generation of F-actin ring. Besides differentiation, autophagy also plays decisive roles in osteoclast function, that the physiological levels of Atg5/7/4B are required for lysosomal [containing H^+^ and cathepsin K (CatK)] trafficking and fusion with the plasma membrane to generate mature ruffled border, as well as to release H^+^ and cathepsin K to resorb bone. During osteoblast differentiation, the binding of BMP2 to its receptors (BMPR) activates Smad signaling pathway to initiate osteogenesis, which also induced the expression of beclin 1 and LAMP2 (autophagy-related proteins) as well as autophagy pathway. Autophagosomes are utilized for transporting mineral crystals to extracellular matrix and thereby facilitating mineralization. Autophagy reduces oxidative stress during osteoblast differentiation via clearance of damaged mitochondria, which also suppresses RANKL production and hence inhibiting osteoclastogenesis. Compared with osteoblast, the autophagy level is increased in osteocyte, which not only maintains homeostasis of osteocyte, but also guarantees a physiological osteocyte-derived regulation on bone remodeling.

Recent studies have identified the importance of autophagy in osteoclast differentiation and function. Autophagy activation has been reported during the osteoclastogenic process. During the RANKL-induced osteoclast differentiation, the autophagic protein levels (such as autophagy related (ATG) 5/7/12) and the LC3-II/LC3-I ratio have been reported to increase in accompany with degradation of p62 (also known as SQSTM1/sequestome1) (47). This degradation plays an essential role in the generation of filamentous actin (F-actin) ring, a key feature of osteoclatogenesis (47, 48). Mutant p62 results in abnormal osteoclasts with increased size, number, multinuclearity, and activity (49). The autophagic proteins Atg5/7/4B and LC3 have also been reported to play decisive roles in regulating the osteoclast-ruffled border (RB) generation and the lysosomal secretion (Figure 1), thereby determining osteoclast function *in vitro* and *in vivo* (50). Especially, in rheumatoid arthritis (RA) patients, autophagy is found activated by the pro-inflammatory cytokine tumor necrosis factor α (TNF- α) in osteoclasts, which results in induced osteoclastogenesis and bone resorption *in vitro* and *in vivo*, suggesting a central role of autophagy in the pathogenesis of inflammatory bone loss (51).

Autophagy has long been considered as a necessary part in cell differentiation (22). In a recent study, induced autophagy has been found during osteoblast differentiation and mineralization *in vitro*; autophagosomes act as cargos to transport the intracellular mineral crystal-like structures to facilitate the extracellular mineralization (52). Autophagy inhibition can result in impaired mineralization *in vitro* and decreased bone mass/volume *in vivo* (Table 1), which is usually followed by induced oxidative stress and RANKL production (52). These results suggest the fundamental roles of autophagy during osteoblast differentiation and mineralization, which serves as mineralization vehicles, protects osteoblast from increased oxidative stress and moreover, reduces osteoblast-derived RANKL production and thereby inhibits osteoclastogenesis during bone formation (52). This is in accordance with the phenomenon that autophagy deficiency/inhibition in osteoblast leads to an osteoporotic-like phenotype with induced osteoclast differentiation (62). Another study found out that bone morphogenetic protein 2 (BMP2), an osteoinductive agent for clinical-use, leaded to increased protein levels of beclin-1 and lysosome-associated membrane protein 2 (LAMP2) (63), both of which are known as autophagy markers (64, 65). On the other hand, autophagy inhibition has been found to affect the differentiation and immunoregulatory capacities of MSCs in ovariectomy-induced osteoporosis model mice (66). In the field of bone tissue-engineering and biomaterials development, the autophagy-inductive bioactive silica nanoparticles have been found to promote osteogenesis (67), suggesting that autophagy could be a potential therapeutic target for bone repair (Figure 1).

**Table 1:** Effects of *Atg* gene-knockout on osteoimmunology *in vivo*.

**Cell type**	***Atg 5*-/-**	***Atg 7*-/-**	**Possible effects on bone resorption**	**Possible effects on bone formation**
Osteoclast	Differentiation↓ (50)	Impaired cathepsin K secretion (53)	Downregulation	Unknown
Osteoblast	Differentiation↓ RANKL production↑ (52)	Differentiation↓ (54)	Upregulation	Downregulation
Osteocyte	Unknown	Aging-like phenotype (54)	Downregulation	Downregulation
M1 macrophage	Polarization↑ (55, 56)	Polarization↑ Aging-like phenotype (57)	Upregulation	Downregulation
M2 macrophage	Polarization↓ (55, 56, 58)	Polarization↓ Aging-like phenotype (57)	Upregulation	Downregulation
Th17 cell	Polarization↑ (59)	IL-17 production↑ (60)	Upregulation	Upregulation
Treg cell	Polarization↓ (61)	Polarization↓ (61) Function↓ (60)	Upregulation	Unknown

Autophagy is particularly critical for terminally-differentiated cells such as neurons and osteocytes, which provides these cells with “intracellular refreshment” to enable the cellular homeostasis and function during their long-life periods (13, 22). Osteocyte is terminally-differentiated osteoblast embedded in bone matrix. The long dendritic processes of osteocyte facilitate the communication within osteocytes as well as the connection with bone surface, making osteocyte capable of sensing mechanical or bio-chemical stimulus from the microenvironment (14). Osteocyte in response to the stimulation therefore acts as director in bone remodeling such as producing RANKL and sclerostin (43, 44, 46, 68). Living in a hypoxic and potentially nutrient poor environment, osteocyte has been reported to keep higher levels of autophagy than the bone surface osteoblast *in vivo* (13, 69). Accordingly, it is found that the terminally-differentiated osteocytes show induced autophagy, as compared with the pre-osteocytes *in vitro* (69). Genetic autophagy suppression (selective *Atg7*-deletion) in murine osteocytes results in skeletal changes in young adult mice (Table 1), such as decreased bone mass and volume, reduced osteoclastogenesis and impaired bone formation, as well as induced ROS in the bone marrow; a phenotype similar to the aging bone (70). Another study has also found that autophagy-activation/inhibition is involved in glucocorticoids-related bone loss (71). All these studies suggest that autophagy at a certain level maintains the homeostasis and function osteocyte to facilitate the physiological balance of bone remodeling (Figure 1).

## Autophagy in Osteoimmunology

### Regulations of the Immune System on Bone Remodeling

The crosstalk between skeletal and immune systems was initially identified by the finding that immune cells-originated interleukin-1 (IL-1) could induce osteoclastogenesis (5). Since then, more evidence has revealed the regulatory role of immune system on bone remodeling (4). T-helper cells, derived from the adaptive immune system, have been found to play a critical role in inflammatory bone loss in arthritis via producing RANKL (72). Besides RANKL, the type 17 helper T (Th17) cell-originated IL-17 has long been identified as a key pre-inflammatory cytokine that promotes osteoclastogenesis (73, 74); while the Th2 cell-originated IL-4 and IL-10 are considered as inhibitors for osteoclasts (75–78). On the other hand, the immune-suppressive regulatory T (Treg) cells (79), inhibit osteoclastogenesis either in a direct cell-to-cell contact-dependent manner (cytotoxic T-lymphocyte-associated protein 4 (CTLA-4) on Treg cells binding with CD80 and CD86 on osteoclast precursors), or via production of IL-4, IL-10, and TGF-β (80, 81). Cells from the innate immune system, such as macrophages, not only serve as osteoclast precursors (82), but also participate in the osteoclastogenesis regulation. Macrophages are a population of cells with three subsets: (1) non-activated M0 macrophage; (2) pro-inflammatory M1 macrophage, which is classically activated by microbe-derived lipopolysaccharide (LPS) or Th1 cells-derived IFNγ; and (3) anti-inflammatory M2 macrophage, which is alternatively activated by Th2 cells-derived IL-4 or IL-13 (83–86). M1 macrophage induces osteoclastogenesis by producing cytokines such as IL-1α/β (87, 88), IL-6 (89–91), TNF-α (92–95); while M2 macrophage reduces osteoclast differentiation via secretion of IL-10 and TGF-β (85, 96, 97).

The regulation of immune system on osteogenesis is not so clear-cut. There are conflicting results regarding the positive/negative effects of inflammatory/anti-inflammatory cytokines on osteoblast differentiation, known as IL-1 (98–101), IL-17 (102–104), TNF-α (100, 101, 105, 106) and IFNγ (107, 108). An interesting finding is that the pro-inflammatory cytokine IL-6 could induce osteogenesis through the oncostatin M (OSM)-STAT3 signaling pathway, suggests the inflammatory response, at a certain level, could initiate osteoblast differentiation (109–115). This is in accordance with the phenomenon that early stage inflammation with macrophage infiltration is regarded as indispensable in bone fracture healing (116). However, this inflammation will be gradually quenched, as the M1 to M2 macrophages conversion happens along with bone repair. This conversion has been found to improve bone formation (116, 117). The M2 macrophage-derived factors, such as BMP2 and TGF-β, are identified to promote osteoblast differentiation and functions, as well as to enhance mineralization (118, 119). These findings suggest that the transformation from the M1 macrophage-mediated inflammatory microenvironment, to the M2 macrophage-mediated regenerative one, should be a required part in bone formation; modulation of M2 polarization should be considered as a potential therapeutic approach for bone regeneration.

### The Role of Autophagy-Mediated Immunomodulation in Bone Remodeling

Autophagy is now identified as a multifunctional pathway in immunity such as lymphocyte differentiation (22), pathogen elimination (120), antigen presentation and inflammation regulation (7, 15). T cell-specific ATG genes deletion (such as *Atg7* or *Atg5*) results in decreased T lymphocyte counts, mitochondria accumulation, and induced apoptosis in mature T cells (121, 122). This is due to the critical role of autophagy-mediated mitochondria clearance in the development of thymocytes into circulating mature T cells (123). Beside differentiation, autophagy also provides barriers against invading pathogens, that autophagosome and autophagolysosome are utilized for selective-detection and elimination of intracellular pathogens (120, 124–127). Especially, the immunomodulatory roles of autophagy have been found in both innate and adaptive immune responses, making autophagy a potential key regulator in osteoimmunology.

The importance of autophagy has long been addressed in macrophage polarization and inflammatory response. Although autophagy is induced by toll-like receptor 4 (TLR4) signaling during M1 macrophage polarization (128), further research has suggested that autophagy plays an immunosuppressive role in macrophage inflammatory response (129). *Atg5*- or *Atg16L1*-deficiency on macrophage is found to direct M2 macrophage to polarize toward a M1-like phenotype with induced secretion of pro-inflammatory cytokines (55, 58). Mice with *Atg5*-knockout macrophages showed induced systemic inflammation (56). Primary bone marrow-derived macrophages (BMDMs) obtained from this mice type exhibited abnormal polarization, that the M1 polarization was increased while the M2 polarization was impaired (Table 1), which further indicating that autophagy-deficiency would induce inflammatory response in macrophages (56). In mice with *Atg 7* gene deletion in the hematopoietic system (vav-*Atg7*-/- mice), monocytes failed to differentiated into macrophages under M-CSF stimulation (130). Moreover, macrophages obtained from vav-*Atg7*–/– mice were found to have a phenotype similar to aged macrophages, which showed reduced abilities of phagocytosis and nitrite burst, while induced inflammatory response (Table 1); suggesting that autophagy maintained at a certain level would correct the abnormities in immune system to prevent aging associated chronical inflammation (57). It has been demonstrated that autophagy inhibition (either by *Atg* gene deletion or pharmacological intervention) results in induced IL-1β secretion of macrophage, suggesting autophagy limits the inflammatory response of macrophage (15, 131, 132). Further studies have found that inflammatory stimulus causes mitochondrial damage, and then consequently results in induced ROS release/apoptosis in macrophage. ROS interact with NF-κB signaling pathway and then activate the NLRP3 inflammasome to trigger the secretion of IL-1β and IL-18, therefore eventually initiating the inflammatory cascade (132–134). During this process, autophagy scavenges the damaged mitochondria through a collaboration between p62 and LC3, that p62 selectively recognizes damaged mitochondria by its UBA domain, which, collectively, combines with LC3 and ensures the lysosomal degradation of damaged mitochondria, thereby interrupts the inflammatory cascade (134–136). As mentioned before, the macrophage inflammation has been demonstrated to induce osteoclastogenesis and bone loss, while the conversion from pro-inflammatory M1 toward anti-inflammatory M2 phenotypes has been suggested to improve bone repair (116, 117). Therefore, this autophagy-mediated regulation on macrophage response should be considered as beneficial for bone regeneration. The nanomaterials-derived autophagy induction has been found to potentially introduce a polarization toward M2 macrophage and thereby improve osteogenesis (137), which further suggests that autophagy could be a potential immunomodulation target in regenerative medicine, especially for therapies against disorders with inflammatory bone loss, such as arthritis (138), periodontitis (139), periapical lesions (140).

Besides its role in the innate immunity, autophagy also acts as a key regulator in the adaptive immune response, such as T cell activation and polarization. Autophagy promotes major histocompatibility complex (MHC) class II-mediated antigen presentation via inducing the fusion of antigens to LC3 in CD4^+^ T cells (141), which facilitates the elimination of autoreactive CD4^+^ T cells (123). On the other hand, although autophagy-dependent antigen presentation is required in in antimicrobial response of dendritic cells (DCs) (141, 142), the autophagy-deficient DCs show hyper-stable interactions with T cells and thereby enhance T cell activation, suggesting the modulatory role of autophagy to prevent excessive T cell response (143). Consistent with this idea, a study has found that graphene quantum dots (GQDs) induce the tolerogenic phenotype of DCs in an autophagy-dependent manner, which show reduced capacity in antigen-presenting and thereby reduce T cell inflammatory response via introducing the polarization of Th1 and Th17 cells toward Th2 and Treg cells, respectively (144). Autophagy also directly inhibits nuclear factor-κB (NF-κB) activation in in antigen-activated T cells and thereby suppresses inflammation (145). On the other hand, autophagy prevents the secretion of macrophage-derived IL-1β (132–134), a cytokine known as promoting Th17 cell response via collaboration with IL-6 and TGF-β (59). In mice with *Atg5*-deficient myeloid cells, CD4^+^ Th17 cell response is induced (59), further suggesting the role of autophagy in preventing inflammation (Table 1). Although inflammatory cytokines such as IL-17 has been reported to induce osteogenesis, excessive IL-17 production results in enhanced RANKL secretion and osteoclastogenesis (73, 74) and therefore is still considered as detrimental for bone regeneration. Especially, autophagy-mediated conversion from Th1 to Th2 cells would in turn induce the polarization from M1 to M2 macrophages (146, 147), a central part in bone regeneration (116, 117). Hence, it could be presumed that autophagy-derived immunomodulation on T cells creates a microenvironment favoring bone repair.

The role of autophagy in osteoimmunology has been further demonstrated in the pathogenesis of autoimmune rheumatic diseases, such as rheumatoid arthritis (RA), a disease with abnormities in organs including joints, heart, vascular system, lungs, and skin (148, 149). Chronical inflammation, as well as bone and cartilage destruction are typical syndromes in RA (150), which mainly due to the interactions within local cells, known as immune cells (such as T and B cells, macrophages), synovial fibroblasts, chondrocytes, as well as osteoclasts and osteoblasts. As autophagy plays decisive roles in osteoclastogenesis, the inhibition of autophagy successfully reduces bone destruction and osteoclast formation in experimental arthritis mouse models (151), suggesting drugs with autophagy inhibition could be used to prevent bone loss in RA patients (148). In addition, autophagy protects cells from apoptosis—a crucial mechanism to extinguish excessive inflammation (132–134, 148), therefore playing essential roles in the pathogenesis and progression of RA, via regulating the balance between immune cell survival and death (148). Induced autophagy/reduced apoptosis have been observed in synovial fibroblasts and synovial tissues obtained from RA patients (152–154), while autophagy inhibition has been found to reduce synovial inflammation in a collagen induced arthritis (CIA) rat model (148, 155). Autophagy hyper-activation has been found in CD4^+^ T cells obtained from CIA mouse, and autophagy is considered to regulate T and B lymphocytes homeostasis to maintain the RA chronical inflammatory response (148, 156). Besides, autophagy dysregulation in endothelial cells is considered as responsible for atherosclerosis in RA (157). Especially, autophagy participates in the pathogenesis of RA via inducing the generation of citrullinated peptides, which consequently interrupting immune tolerance (150). The anti-cyclic citrullinated peptide (anti-CCP), an autoantibody (against citrullinated peptides) produced by immune cells upon activation of self-antigens, is a general marker for clinical RA diagnosis (150, 158, 159). The RA patient-derived anti-CCP antibody (Ab) has been found to induce osteoclastogenesis and bone loss (160). Autophagy is recently considered to participate in both the presentation of citrullinated peptides and the generation of anti-CCP Ab (148). Autophagy is required for the antigen presenting cells (APCs) to perform the presentation of citrullinated proteins (161). Furthermore, autophagy is involved in the citrullination processes of Normal Human Bronchial Epithelial (NHBE) cells and human synovial fibroblasts (150, 162). Increased citrullinated peptide production has been observed following autophagy-induction in human synovial fibroblasts from RA patients, and the autophagy level is significantly associated with that of anti-CCP Ab in early-stage RA patients (150), suggesting the fundamental role of autophagy in RA establishment via inducing the generation of citrullinated peptides (150, 163). All these studies indicate that autophagy-derived modulation on osteoimmunology plays a central part not only in physiological bone homeostasis but also in pathological bone diseases, which needs further study in the future.

## Future Remarks & Conclusion

**Figure 2:**
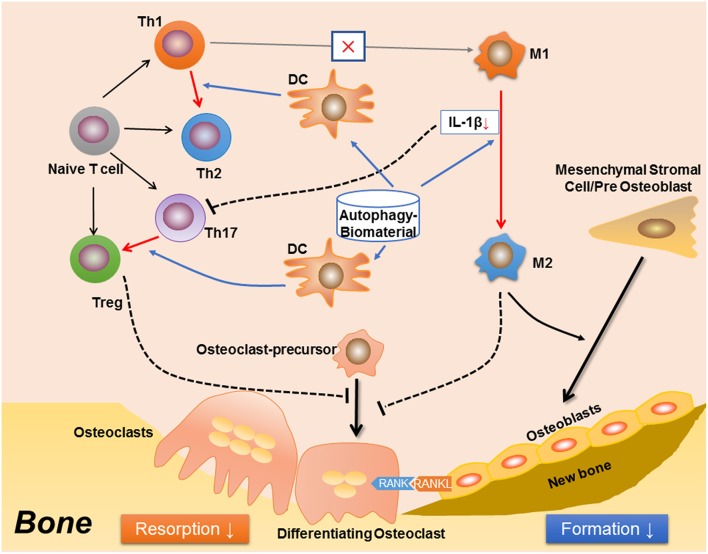
A proposed effect in osteoimmunology regarding biomaterial with the property of inducing autophagy (autophagy-biomaterial). The autophagy-biomaterial should induce bone repair by suppressing the inflammatory response. On one hand, autophagy-induction directly reduces macrophage inflammation and IL-1β secretion. The decreased IL-1β also impedes the polarization and function of Th17 cell (Th17). On the other hand, autophagy-induction in dendritic cell (DC) interrupts its interaction with T cells, thereby inhibiting T cell inflammatory response via introducing the polarization from Th1 toward Th2 cells, as well as that from Th17 to Treg cells. The Th1 to Th2 conversion would in turn reduce M1 polarization while induce M2 polarization. Hence, the autophagy-biomaterial creates an immune microenvironment favoring bone regeneration: the limited inflammatory responses of T cell and M1 macrophage reduce osteoclastogenesis, while the conversion of M1 to M2 macrophages improves osteogenesis.

Many questions still remain un-resolved regarding the role of autophagy in osteoimmunology. For example, although autophagy is indispensable in osteoclastogenesis, rapamycin (also named as sirolimus), an autophagy inducer via inhibition of the Ser/Thr protein kinase mTOR (mammalian target of rapamycin) (164, 165), has been found to reduce osteoclastogenesis and bone resorption in a mouse model of arthritis, an effect similar to anti-TNF (by Infliximab) treatment (166). It is also found that rapamycin reduces osteoclastogenesis in young rats (167) and post-transplant bone resorption in renal transplant patients (168). This is quite contrary to the positive effect of autophagy in osteoclast differentiation and function as mentioned before. It is presumed that autophagy plays a more maintenance than regulatory role in the differentiation of osteoclast, which is induced and kept in a certain level in response to energy/metabolism variations or intracellular accumulation of damaged organelles such as mitochondria. Whereas, in immune response, autophagy might act more as a regulator to quench the inflammation fire (7), which in turn reduce bone resorption (Figure 2). The rapamycin-mediated inhibition of bone loss might be achieved via immunomodulation, suggests that autophagy is an attractive target for osteoimmunology regulation to improve bone tissue regeneration (Figure 2). Autophagy might also participate in the mutual regulations between immune-skeletal systems. Previous studies have suggested that the immunosuppressive role of MSCs is achieved through the programmed death 1/ programmed death-ligand 1 (PD-1/PDL1) (169), an autophagy-related signaling pathway (170). Another study has found that the autophagy regulator p62 plays a central role in maintenance of the “macrophage-osteoblast niche,” which is indispensable for the retention of HSCs in bone marrow (171). It is also found that compared with the undifferentiated MSCs, the osteogenically differentiated MSCs not only induce the recruitment of macrophages, but also regulate local macrophage response in a VEGFA–C-X-C motif chemokine 12/C-X-C chemokine receptor type 4 (VEGFA-CXCL12/CXCR4) axis dependent manner (172). As CXCR4 signaling has been reported to regulate autophagy via the cross-talk with mTOR (173–175), hence, autophagy should be involved in MSC-mediated regulation on immune cells. Furthermore, It could be predicted that immune cells, especially macrophage, regulate bone remodeling in an autophagy-dependent manner, as both the M1 and M2 macrophage-derived cytokines have been found to modulate autophagy, such as IL-1 (176), TNF-α (177–179), IL-10 (180–182), TGF-β (183, 184); the fundamental role of M1-M2 conversion in osteogenesis might be partially due to different autophagy levels and metabolism states during the differentiation from osteoblast to osteocyte.

In summary, autophagy, a conserved “self-eating” pathway present in all mammalian cells, plays a complex role in osteoimmunology, that at one level, autophagy maintains the cellular homeostasis during differentiation of osteoclast and osteoblast, facilitates the survival and function of osteocyte, and more importantly, direct the immune response to avoid the damage of excessive inflammation. Therefore, the definition of autophagy as “self-eating” should be more appropriate as “self-editing” in maintaining bone homeostasis. This regulatory role in osteoimmunology suggests autophagy could be a novel therapeutic target (e.g., autophagy-inductive biomaterial) to improve bone regeneration in the future translational medicine (Figure 2).

## Author Contributions

All authors listed have made a substantial, direct and intellectual contribution to the work, and approved it for publication. LX: involved in the concept and design of the article and wrote the manuscript. YX: involved in the conception and design of the article and reviewed the manuscript.

### Conflict of Interest Statement

The authors declare that the research was conducted in the absence of any commercial or financial relationships that could be construed as a potential conflict of interest.
